# Heavy Metals Presence in the Soil and Their Content in Selected Varieties of Chili Peppers in Slovakia

**DOI:** 10.3390/foods10081738

**Published:** 2021-07-28

**Authors:** Judita Lidiková, Natália Čeryová, Marek Šnirc, Janette Musilová, Ľuboš Harangozo, Alena Vollmannová, Jan Brindza, Olga Grygorieva

**Affiliations:** 1 Department of Chemistry, Faculty of Biotechnology and Food Sciences, Slovak University of Agriculture in Nitra, Tr. A. Hlinku 2, 949 76 Nitra, Slovakia; xceryova@uniag.sk (N.Č.); marek.snirc@uniag.sk (M.Š.); janette.musilova@uniag.sk (J.M.); lubos.harangozo@uniag.sk (Ľ.H.); alena.vollmannova@uniag.sk (A.V.); 2 Department of Genetics and Plant Breeding, Faculty of Agrobiotechnology and Food Resources, Slovak University of Agriculture in Nitra, Tr. A. Hlinku 2, 949 76 Nitra, Slovakia; jan.brindza@uniag.sk; 3 Department of Fruit Plants Acclimatisation, National Botanical Garden of the National Academy of Sciences of Ukraine, Timiryazevska 1, 04014 Kyiv, Ukraine; olgrygorieva@gmail.com

**Keywords:** heavy metals, *Capsicum*, chili, food safety, cadmium

## Abstract

*Capsicum* chili peppers are popular vegetables in Slovakia. They provide a supply of health-promoting substances, but contaminated vegetables can pose a serious health risk to the people who consume them. Therefore, the aim of this study was to determine the content of heavy metals (Mn, Zn, Cr, Cu, Ni, Cd, Pb and Hg) in the soil as well as in selected varieties of the genus *Capsicum* grown in southern Slovakia. The results were compared with the limit values given by the Law no. 220/2004 (valid in the SR) as well as threshold values proposed by the European Commission (EC) (2006). The gained result showed that the total content of Cd (1.64 mg/kg) as well as the available mobile forms of Cd (0.12 mg/kg) and Pb (0.26 mg/kg) was exceeded on the soil on which *Capsicum* cultivars were grown. The limit values of other monitored heavy metals (Mn, Zn, Cr, Cu, Ni, and Hg) were not exceeded. The studied species of the genus *Capsicum* did not accumulate monitored heavy metals. It can be stated that consumption of chili peppers does not pose any risk for human health.

## 1. Introduction

Soil contamination with heavy metals has increased over the last few decades due to the burning of fossil fuels, municipal waste disposal, mining and smelting, as well as the application of pesticides, fertilizers and wastewater [1], including protected areas [2]. According to the FAO, heavy metals are non-biodegradable pollutants, characterized by high persistence in ecosystems and the ability to accumulate in the food chain [3,4], whose already low concentrations can seriously damage the health of soils, plants, animals, and people [5,6]. For example, the Lowest Observed Adverse Effect Level (LOAEL) for Hg is 0.63 mg/kg/day, and the Pb blood level of ≥300 µg/L in children and adults is associated with slowing of nerve conduction velocity [7,8]. Heavy metals, such as Cr, Cd, Hg, Pb and As, have been identified by the United States Environmental Protection Agency (USEPA) as a priority pollutant due to their persistence and irreversible toxic properties [9]. These metals can induce oxidative stress by generating free radicals and reducing antioxidant levels. The accumulation of heavy metals in the human body can lead to organ toxicity; they can especially have an effect on the gastrointestinal tract, and on the nervous, respiratory and reproductive systems [10,11]. Children may be particularly susceptible to heavy metal intoxication, which may adversely affect normal growth [12].

Lead (Pb) and cadmium (Cd) are common risk elements found in contaminated soil. Pb is one of the least mobile soil elements. Exposure to Pb may affect human health. Long-term exposure causes low IQ, and impaired neurobehavioral development and growth of children [13], cardiovascular problems, renal dysfunction, and may have genotoxic effects [14]. Cd is one of the most mobile and potentially bioavailable soil elements. It is used in many industrial processes such as the production of nickel–cadmium batteries, solar cells and electroplating [15]. It affects plant germination and growth, affects the rate of photosynthesis and reduces chlorophyll content [16,17]. In humans, Cd is reported to be carcinogenic [18]; epidemiological data point to Cd as a risk factor for lung, bladder and prostate cancer [19,20]. Cd induces cancer through several mechanisms, induces oxidative stress, inhibits apoptosis, damages DNA and inhibits DNA repair [21].

Unlike Cd and Pb, some metals such as Zn, Mn, Cu and Ni belong to micronutrients, but at elevated concentrations their toxic effects may occur; e.g., Cu is required for enzymes such as tyrosinase and superoxide dismutase and plays an important role in the development of the central nervous system [22]. Zinc (Zn) is required for protein function and activity, for DNA synthesis as well as playing a role in male fertility [23], and in general it is one of the microelements (together with Se) very rare in food and plants. Some researchers suggested starting cultivation, in cooperation with farmers, of some wild species, e.g., Aegilops ventricosa Tausch that, unlike cultivated wheat varieties, has a higher quantity of microelements, such as Zn, verifying the prospect of the production and marketing of its flour and/or pasta as a natural alternative to conventional medicine, and being helpful for people with Zn deficiencies [24,25]. Zn is an essential micronutrient, as it affects the activity and production of hundreds of different enzymes (dehydrogenases, peptidases, proteinases). It acts as a cofactor in enzymatic reactions involved in DNA expression, playing an important role in membrane stabilization and vitamin A metabolism [26]. High levels of Zn in the human body can disrupt the homeostasis of other essential elements. Large doses of Zn can interfere with lipoprotein metabolism. The long-term increased intake of Zn reduces the absorption of Cu and can cause its deficiency in the body. Zn has a relatively low toxicity and the serious effect of Zn intoxication on human health is a relatively rare phenomenon [27]. Acute Zn poisoning is manifested by vomiting, diarrhea and fever. For humans, Zn is not teratogenic or mutagenic.

Manganese (Mn) is a micronutrient needed in small amounts for human growth, development, and functioning, it is a cofactor of many enzymes (glutamine synthetase, arginase, pyruvate carboxylase) but in excessive amounts it can have strong neurotoxic effects. The neurotoxicity of Mn is associated with cognitive and motoric disorders, known as manganism. The exact underlying mechanism of Mn toxicity is unknown, but the clinical signs are identical to Parkinson’s disease [28].

Chromium (Cr) is a naturally occurring heavy metal that occurs in the environment as Cr^3+^ and Cr^6+^. Cr^6+^ can persist in soil or sediment for years, especially if the soils are sandy or contain low levels of organic matter [29]. Cr can be released into the air and drinking water from industrial processes. The reduction in Cr^6+^ to Cr^3+^ leads to the formation of reactive products that contribute to the cytotoxicity, genotoxicity and carcinogenicity of Cr^6+^-containing compounds [30]. Unlike Cr^6+^, Cr^3+^ is considered a micronutrient in humans, as it is essential for the metabolism of sugars and lipids. High doses of Cr and its long-term exposure can lead to various cytotoxic and genotoxic reactions that affect the body’s immune system. Studies have shown that Cr^6+^ induces oxidative stress through the increased production of reactive oxygen species (ROS), leading to damage to genomic DNA and the oxidative degradation of lipids and proteins [31]. Cr^6+^ is an epithelial irritant, has genotoxic properties and is also considered a human carcinogen [32]. Copper (Cu) is an important cofactor in various cellular processes; it is essential for physiological processes such as Fe homeostasis [33], for the biosynthesis of neurotransmitters [34] and for energy metabolism, but increased content can cause various diseases in the human body. The absorption, uptake and transport of Cu are strictly regulated because too much and too little Cu is associated with oxidative cell damage, impaired immune function, and causes organ dysfunction. Cu imbalance is also associated with chronic liver disease, which comes from viral hepatitis infection or other liver damage. Cu toxicity can result in Wilson’s disease. This rare disease is caused by a mutation in the gene and is characterized by excessive Cu accumulation [35]. Soluble Cu represents only a very small part of the total content of Cu in the soil. Cu has a high affinity for organic substances in the solid phase and therefore is not easily leached and can accumulate in surface soil.

Nickel is among the essential micronutrients utilized by plants, but high levels may be highly phytotoxic. Ni induces Fe and Zn deficiency and prevents the absorption of other heavy metals such as Cd, Cr and Pb [36]. The major toxic effects of Ni on humans include allergic contact dermatitis for nickel, airway carcinogenicity, and reproductive toxicity [37].

The Hg^2+^ form of mercury plays a key role in the toxicology of this metal. High levels of this form have strong phytotoxic effects when present in toxic concentrations, they can cause visible injuries and physiological disorders in cells, which cause the production of ROS (reactive oxygen species), which in turn leads to cell disruption. Hg has a high affinity for biomolecules containing sulfhydryl (SH) groups [38]. The mechanism of Hg phytotoxicity may be through the displacement of metal ions from molecules (chlorophyll magnesium), the induction of changes in membrane and organelle permeability, and the inactivation of proteins [39]. High doses of Hg can have serious damaging effects on the developing nervous system, which can result in irritability, behavioral changes, tremors, headaches, hearing and cognitive impairment, dysarthria, and in changes to coordination. Hg is also toxic to the cardiovascular, immune, and reproductive systems. In the cardiovascular system, Hg causes hypertension in humans and animals, which has broad consequences, including changes in endothelial functions [38]. Inorganic Hg can cause kidney failure and damage to the gastrointestinal tract.

The transfer of heavy metals from contaminated soil to edible and consumed vegetables can compromise the quality of edible vegetables, food safety, and is a major route of human exposure to these metals [40].

Chili peppers (*Capsicum annuum* L., *C*. *chinense* L., *C*. *baccatum* L., *C*. *frutescens* L.) belong to the family *Solanaceae* and are grown in many parts of the world as important commercial crops (4.26 millions of tonnes produced worldwide in year 2019.) They are distinguished for their spicy and burning flavor due to presence of capsaicin. They originated in Mexico and other Central American areas, from where they spread throughout the world in the 17th century. Chili peppers are very popular, and are grown and consumed in Slovakia, especially in southern Slovakia. According to FAOSTAT, the production of chilies and peppers in Slovakia in 2019 was 4740 tonnes. These spicy varieties are characterized by high vitamin C and polyphenol content, capsaicinoid content as well as high antioxidant activity, and are an integral part of traditional cooking [41,42]. Due to the high worldwide consumption of various species of the genus *Capsicum* as well as the use of capsaicin as a food additive and its current medical use, the monitoring and evaluation of toxic substances such as heavy metal accumulation in selected varieties of *Capsicum* is very important [43].

A study showed the genotoxic effect of Cd in pepper (*C*. *annuum* L.) as well as the excessive formation of superoxide radicals, which led to oxidative stress and increased lipid peroxidation in *Capsicum* tissues [44]. In another study, Pb showed a significant effect on cytomorphological traits in chili plants, and higher concentrations of Pb showed a genotoxic and mutagenic effect [45]. The consumption of vegetables as well as peppers of the genus *Capsicum* provide health benefits, but heavy metal content above the health-based guidance in contaminated vegetables can pose a serious health risk to the millions of people who consume them. Therefore, it is important to assess these health risks. The risk assessment consists of hazard identification, hazard characterization, exposure assessment, and risk characterization [46]. Food legislation in the European Union includes both hazard- and risk-based approaches for ensuring safety. In hazard-based approaches, simply the presence of a potentially harmful agent at a detectable level in food is used as a basis for legislation and risk management action. Risk-based approaches, on the other hand, try to establish health-based guidance values for human exposure to chemicals, such as acceptable or tolerable daily intakes, using toxicological data; estimates of human exposure are then compared with the health-based guidance value to assess whether there may be an unacceptable risk to health and whether risk management action is needed. Hazard- and risk-based approaches have a common element in that the identification of the hazard is a first step in both. In hazard-based approaches, the hazard may then be characterized. In risk-based approaches, this will be followed by exposure assessment and the integration of exposure with hazard characterization in the final risk characterization step, in order to provide an overall risk assessment, from which to conclude on safety [47].

Therefore, the aim of this study was to determine the content of heavy metals (Mn, Zn, Cr, Cu, Ni, Cd, Pb and Hg) in the soil as well as in selected varieties of the genus *Capsicum* grown in southern Slovakia and potential correlations among content of heavy metals in soil and content of heavy metals in studied species of genus *Capsicum*.

## 2. Materials and Methods

### 2.1. Study Location and Sample Collection

**Figure 1:**
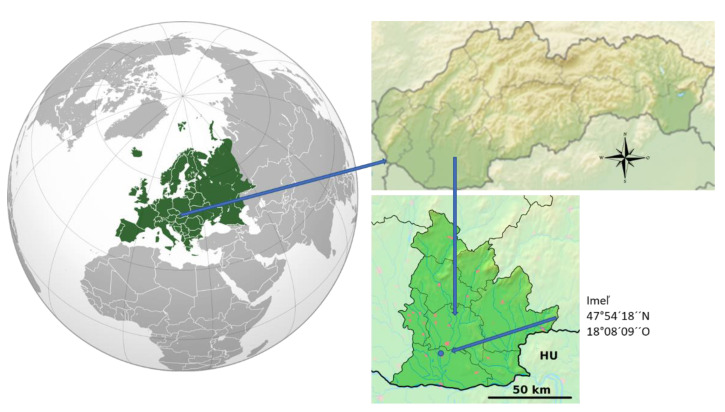
Map of small parcel experiment. Reproduced from [48], with permission from author, 2010, and from [49], with permission from author, 2010.

A small plot experiment was realized in the cadastral area of the village Imeľ. The village of Imeľ is located in southwestern Slovakia, lying in the Danubian Lowland between the rivers Nitra and Žitava (Figure 1). The territory of the village lies at an altitude of 108–121 m. The growing area belongs to the dry, warm climate zone (the average annual air temperature is 9.9 °C, and average annual rainfall is 550 mm). The land in the cadastral territory of the municipality of Imeľ consists mainly of black soils, which belong to the fertile lands, black earth soils, gley black soils, and modal carbonates. In flooded areas, fluvials are modal to gley. In the village, there are sandy soils suitable for growing chili peppers.

Eight samples of *Capsicum (C*. *annuum* L. and *C*. *chinense* L.), namely Sigaretta di Bergamo, Cayenne Long Slim, Chupetinho, Candlelight, Violet Cables, Scotch Bonet Yellow, Bhut Jolokia Red, Bhut Jolokia White, were obtained in this area. The investigated samples of *Capsicum* cultivars were conventionally cultivated in the same locality and under the same conditions. Only NPK fertilization was used to achieve a favorable content of macro elements in the soil.

The varieties of the chili peppers monitored were harvested by hand, after reaching full maturity in the growing season of the plant. The design of the experiment featured 10 m^2^ parcel for each cultivar. A total of 3 kg of representative sample were taken from 4 random places of every parcel. The samples were then cleaned, dried and homogenized, and used for analyses.

The determination of chili pepper dry matter was performed by drying at 105 °C to constant weight (WTC Binder, Tuttlingen, Germany). The samples were dried and afterwards homogenized on the grinder IKA A10 (IKA GmBH, Staufen, Germany). Prior to analyses, homogenized samples were stored in plastic bags. Soil samples were taken from 4 random places from the arable layer (0–20 cm) with a pedological probe GeoSampler (Bürkle GmbH, Bad Belllingen, Germany). After collection, the soil samples were temporarily stored in plastic resealable bags. The soil samples were air dried at room temperature for 2 weeks, under laboratory conditions. They were then cleaned of coarse particles and sieved through a sieve (fine soil I—2 mm), which was used to determine agrochemical characteristics. Fine soil II (0.125 mm) was used to determine the heavy metal content.

### 2.2. Chemical Analysis of the Soil

In each soil sample (4 samples for each analysis), the micronutrient content (Ca, Mg, K, P) by Mehlich II, the exchangeable reactions (pH/CaCl_2_), and the organic carbon and humus content were determined [50,51]. Mobile forms of heavy metals (Mn, Zn, Cr, Cu, Ni, Cd Pb) and total heavy metal content (Mn, Zn, Cr, Cu, Ni, Cd, Pb, Hg) were also determined.

#### 2.2.1. Determination of (pH/CaCl_2_)

A total of 20 g of soil sample and 50 mL of CaCl_2_ (Sigma-Aldrich, Sigma-Aldrich, Inc., St. Loius, MO, USA) solution (c = 0.01 mol/dm^3^) were mixed in a 100 mL plastic bottle and the substances were shaken for 20 min in a horizontal shaker Unimax 2010 (Heidolph Instrument, GmbH, Schwabach, Germany). The samples were then filtered through quantitative filter paper Filtrak 390 (Munktell, GmbH, Bärenstein, Germany) and pH was determined with a pH meter Metrohm 691 (Metrohm, AG, Herisau, Switzerland).

#### 2.2.2. Determination of Humus Content and Content of Organic Carbon (Cox) in Soil

A total of 1 g of fine soil II sample was mixed with 0.1 g of Ag_2_SO_4_ and 10 mL of a prearranged chromium–sulfur mixture. The flasks were heated to 150 °C for 20 min, during which the color changed from brown to yellowish orange. After cooling, samples were titrated with Mohr’s salt solution (c = 0.1 mol/dm^3^) using an indicator (diphenylamine) [50,51].

#### 2.2.3. Determination of Available Micronutrients (Ca, Mg, K, P) in Soil by Mehlich II

At first, the Mehlich II solution was prepared. A total of 11.5 mL of glacial acetic acid was added to 10.7 g of NH4Cl; 0.56 g NH4F and 1 mL concentrated HCl into a glass volumetric flask (1000 mL) and deionized water was added up to the mark. Amounts of 5 g of fine soil I and 50 mL of Mehlich II were weighed into a 100 mL plastic bottle, and mixed. The bottle was then shaken for 10 min on a horizontal shaker Unimax 2010 (Heidolph Instrument, GmbH, Schwabach, Germany). The samples were then filtered through quantitative filter paper Filtrak 390 (Munktell, GmbH, Bärenstein, Germany). The content of micronutrients (Ca, Mg, K,) was determined by flame atomic absorption spectrometry—F-AAS—on a spectrometer SpectrAA 240FS (Varian Inc., Mulgrave, VIC, Australia). The phosphorus content was determined by pipetting 1 mL of the filtrate into a flask, adding 8 mL of solution B and making up the volume to 50 mL with deionized water. After 2 h of staining, the phosphorus content was determined by the Ultraviolet-visible scanning spectrometer Shimadzu UV-VIS 1800, λ = 666 nm, (Shimadzu Corporation, Kyoto, Japan) [52].

#### 2.2.4. Determination of Total Heavy Metal Content in Soil

The total heavy metal content was determined after mineralization in a 2.5 mL 65% HNO3 Suprapur^®^ (Merck, Darmstadt, Germany) and 7.5 mL 37% HCl Suprapur^®^ (Merck, Darmstadt, Germany) mixture. This mixture is able to extract almost all heavy metals from the soil solution, with the exception of silicate and aluminosilicate soil grid structures.

The mineralization tubes were sealed and placed in a microwave digestion apparatus MarsX-press5 (CEM Corp., Matthews, NC, USA). The samples were filtered through quantitative filter paper Filtrak 390 (Munktell, GmbH, Bärenstein, Germany) and diluted with deionized water (0.054 µS/cm). For all procedures, high purity analytical reagents were used. The total heavy metal content was determined using the atomic absorption spectrometer SpectrAA 240FS (Varian Inc., Mulgrave, VIC, Australia) (Mn, Zn, Cu, Cr, Ni) and the atomic absorption spectrometer SpectrAA 240Z (Cd and Pb) with Zeeman background correction. CertiPUR^®^ (Merck, Darmstadt, Germany) calibration standard was used for calibration of the instruments.

The total Hg content was determined by the CV-AAS method on a selective Hg analyzer AMA-254 (Altec, Praque, Czech Republic.)

#### 2.2.5. Determination of Mobile Forms of Heavy Metals in Soil

Mobile (available) forms of heavy metals, which are more accessible for plants, were determined by extracting 20 g of dried soil samples in 50 mL of NH_4_NO_3_ (Sigma-Aldrich, Inc., Saint-Loius, MO, USA) (c = 1 mol/dm^3^) using a horizontal shaker Unimax 2010 (Heidolph Instrument, GmbH, Schwabach, Germany) for 2 h. After extraction, the samples were filtered through quantitative filter paper Filtrak 390 (Munktell, GmbH, Bärenstein, Germany).

The content of mobile forms of heavy metals in soil was determined using the atomic absorption spectrometer SpectrAA 240FS (Varian Inc., Mulgrave, VIC, Australia) (Mn, Zn, Cu, Cr, Ni) and atomic absorption spectrometer SpectrAA 240Z (Cd and Pb) with Zeeman background correction. CertiPUR^®^ (Merck, Darmstadt, Germany) calibration standard was used for the calibration of the instruments.

The measured concentrations of selected heavy metals in soil samples were compared with Slovakian limit values, as given by Act No 220/2004, as well as with Threshold value given by the European Commission (2006).

### 2.3. Chemical Analysis of the Plant Material

Homogenized samples were mineralized in a mixture of 5 mL of HNO_3_ (Suprapur^®^, Merck, Darmstadt, Germany) and 5 mL of deionized water (0.054 µS/cm) in the Mars Xpress 5 closed microwave digestion system (CEM Corp., Matthews, NC, USA). Digestive conditions for the microwave system used included heating at 160 °C for 15 min and maintaining it at a constant temperature for 10 min. The digested material was then filtered through quantitative filter paper Filtrak 390 (Munktell, GmbH, Bärenstein, Germany) and filled to a volume of 50 mL with deionized water. Samples were analyzed by the atomic absorption spectrometer SpectrAA 240FS (Mn, Zn, Cu, Cr, Ni) (Varian Inc., Mulgrave, VIC, Australia) and atomic absorption spectrometer SpectrAA 240Z (Cd and Pb) with Zeeman background correction. The limit of detection for Mb, Zn, Cu, Cr, Ni was set at 3.0; 6.0; 2.0; 3.0; 8.0 μg/kg, respectively, and for Cd and Pb 10.0, 10.0 ng/kg, respectively. The limit of quantification for Mn, Zn, Cu, Cr, Ni was set at 9.0; 18.0; 6.0; 9.0; 24.0 μg/kg, and for Cd and Pb 30.0 and 30.0 ng/kg, respectively.

The total Hg content was determined by the CV-AAS method on a selective Hg analyzer AMA-254 (Altec, Praque, Czech Republic) in all types of dried and homogenized samples. The limit of detection for Hg was set at 1.5 ng/kg dry weight (DW) and the limit of quantification at 4.45 ng/kg DW [53].

The content of heavy metals determined in plant samples were evaluated according to Decree no. 2/1994 Coll. Decree of the Ministry of Health of the Slovak Republic and maximum values according to Commission Regulation 1881/2006 (EC).

A description of analytical parameters for the determination of the elements is described in Table 1.

### 2.4. Environmental and Health Risk Assessment

We used the following parameters to evaluate the contamination of the soil where the monitored chili pepper varieties were grown:

The contamination factor $\left(C_{f}^{i}\right)$ is a quantification of the degree of contamination relative to either average crustal composition of a respective metal or to the measured background values from geologically similar and uncontaminated area [54]. It is expressed as a ratio of total content of heavy metal in soil ($C_{s}^{i}$) and their background values (C_RefS_) [55]

(1) $$C_{f}^{i}=\left(\frac{C_{s}^{i}}{C_{RefS}}\right)$$

For the calculation of $C_{f}^{i}$, the background values from soil monitoring of the Slovak Republic [56] were used.

Based on the contamination factor, soil is characterized as follows: $C_{f}^{i}$ < 1: low contamination, 3 < $C_{f}^{i}$ < 6: considerable contamination, $C_{f}^{i}$ > 6: very high contamination [55].

The degree of contamination (C_deg_) is the sum of contamination factors for all monitored risk elements and is calculated based on the relationship:

$$C_{deg}=\sum^{}C_{f}^{i}$$

The sum of $C_{f}^{i}$ for all metals represents an integrated degree of environmental pollution [57,58]. Based on the degree of contamination, soil is characterized as it follows: C_deg_ < 5: low contamination, 5 C_deg_ < 10: moderate contamination, 10 ≤ C_deg_ < 20: considerable contamination, C_deg_ ≥ 20: high contamination [55].

The potential ecological risk factor ($E_{r}^{i}$) is used to evaluate the toxicity of the monitored elements. Its calculation is based on the relationship:

$$E_{r}^{i}=T_{r}^{i}\times C_{f}^{i}$$

$T_{r}^{i}$—the biological toxic factor of each element. In particular, the toxic factor should provide information on potential modes of transport of toxic substances to humans and on human hazards [30]. Based on the potential ecological risk factor, soil is characterized as follows: $E_{r}^{i}$ < 40: low risk, 40 ≤ $E_{r}^{i}$< 80: moderate risk, 80 ≤ $E_{r}^{i}$ < 160: considerable risk, 160 ≤ $E_{r}^{i}$ < 320: high risk, $E_{r}^{i}$ > 320: very high risk [57].

The index of geoaccumulation (I_geo_) is used to quantify the degree of the contamination of an individual element. It is calculated according to the formula:

$$I_{geo}=log_{2}\left(\frac{C^{i}}{1.5\times B^{i}}\right)$$

where C^i^ is the concentration of heavy metal in soil and B^i^ the background values from soil monitoring of the Slovak Republic [56]. Soil contamination on the basis of soil using the index of geoaccumulation is characterized as follows: I_geo_ = 0: no contamination, 0 ≤ I_geo_ ≤ 1: light contamination, 1 ≤ I_geo_ ≤ 2: slightly moderate contamination, 2 ≤ I_geo_ ≤ 3: moderate contamination, 3 ≤ I_geo_ ≤ 4: slightly heavy contamination, 4 ≤ I_geo_ ≤ 5: heavy contamination, I_geo_ ≤ 5: extremely heavy contamination [59].

The pollution load index (PLI) serves to assess soil quality in terms of hazardous risk elements. It is defined as the nth root of the product of contamination factors ($C_{f}^{i}$) [58,60], and is calculated according to the relation:

$$PLI={\left(C_{f1}^{i}\times C_{f2}^{i}\times C_{f3}^{i}\times \ldots \times C_{fn}^{i}\right)}^{\frac{1}{n}}$$

The bioaccumulation factor (BAF) is defined as the ratio of the content of the monitored heavy metal in the plant material in relation to the content in the soil [61], and reflects the ability of the plant to absorb heavy metal. The BAF was calculated as follows:

$$BAF=\frac{total\;heavy\;metal\in dried\;plant\;materials}{total\;heavy\;metals\in dried\;soil\;samples}$$

The provisional tolerable intake (PMTDI, PTWI, PTMI) estimates the amount per unit body weight of a potentially harmful substance or contaminant in food or water that can be ingested over a lifetime without risk of adverse health effects. JECFA uses the term PTWI, or provisional tolerable daily intake, for contaminants that may accumulate in the body. The weekly designation is used to stress the importance of limiting intake over a period of time for such substance [62]. The provisional tolerable weekly intake (PTWI) is the end-point used by the Joint FAO/WHO Expert Committee on Food Additives for food contaminants such as heavy metals with cumulative properties. Its value represents the permissible human weekly exposure to those contaminants unavoidably associated with the consumption of otherwise wholesome and nutritious foods.

The provisional tolerable monthly intake (PTMI) is an end-point used by the Joint FAO/WHO Expert Committee on Food Additives for a food contaminant with cumulative properties that has a very long half-life in the human body. Its value represents the permissible human monthly exposure to a contaminant unavoidably associated with otherwise wholesome and nutritious foods. The provisional maximum tolerable daily intake (PMTDI) is the reference value, established by the Joint FAO/WHO Expert Committee on Food Additives, used to indicate the safe level of intake of a contaminant with no cumulative properties. Its value represents permissible human exposure as a result of the natural occurrence of the substance in food and drinking water. In the case of trace elements that are both essential nutrients and unavoidable constituents of food, a range is expressed, the lower value representing the level of essentiality and the upper value the PMTDI. The tolerable intake is generally referred to as “provisional”, as there is often a paucity of data on the consequences of human exposure at low levels, and new data may result in a change to the tolerable level [63].

The daily consumption of chili pepper can be as high as 15 g per person in Mexico and Korea. At the other end of the spectrum are the Northern European countries where the daily chili pepper consumption is less than 1 g per person [64]. According to FAOSTAT, consumption of green peppers and chilies in Slovakia represent 12 g/capita/day. The estimated chili pepper consumption in Slovakia is 1 g/day/per capita.

The tolerable intakes for Cd, Pb, Hg, Cu and Zn is established by WHO.

The limit values of (Zn, Ni, Cu, Cd, Pb, Hg) for soil was evaluated according to legislation valid in the Slovak Republic (Act number 220/2004) together with the threshold value of (Zn, Ni, Cu, Cd, Pb) according to the European Commission (2006).

The limit values of (Zn, Cu, Cr, Ni, Cd, Pb, Hg) in plant matter was evaluated according to the Food Codex of the Slovak Republic, and the maximal level of (Cd, Pb) according to Commission Regulation 1881/2006(EC).

### 2.5. Statistical Analysis

At first, all variables were tested for normality. All the tested variables did not follow the normal distribution according to the Shapiro–Wilk test and Kolmogorov–Smirnov test; therefore, Kruskal–Wallis (nonparametric ANOVA) and Wilcoxon tests were performed to find the significant differences between the tested variables. For a better understanding and interpretation of the results, each cultivar was compared with the median value (horizontal line) using the Wilcoxon test. The Spearman correlation test at the significance level α = 0.05 was used to analyze the relationships between the variables. Principal component analysis was performed to summarize and to visualize the information in a dataset. Descriptive statistics, normality tests, and the principal component analysis were performed using MS Excel with the XLSTAT package [65]. Kruskal–Wallis and Wilcoxon tests were performed in RStudio software, version 1.2.5033 [66].

## 3. Results and Discussion

### 3.1. Agrochemical Characteristics, Contents of Nutrients and Heavy Metals in Soil

Agrochemical characteristics, and contents of nutrients in soil from the cultivation locality Imeľ are presented in Table 2.

The pH of the soil of the monitored land has an average value of 7.55 ± 0.07, which can be characterized as alkaline and moderately humic, with humus content of 3.10%. The soil was characterized by a medium content of potassium and a very high content of phosphorus and magnesium (P = 174 mg/kg, Mg = 319 mg/kg).

In this work, we determined the total content of heavy metals in the soil and compared the values of heavy metals with the limit value—for a soil extract by *aqua regia* according to legislation valid in the Slovak Republic (Act number 220/2004) as well as with the threshold value—European Commission (2006). The higher content of heavy metals in the soil could be reflected in the quality of cultivated crops, followed by accumulation in the aboveground mass. Based on their physiological purpose, heavy metals are divided into non-essential and essential heavy metals. While essential heavy metals (Fe, Cu, Zn, Mn) are relatively less toxic and in certain concentrations are needed for healthy plant growth and development, non-essential heavy metals (Cd, Pb, As, Hg) are highly toxic for plants and humans, even at low-level concentrations [9]. The total heavy metal content in the soil from the crop growing locality Imeľ is presented in Table 3.

We can state that out of the total content of heavy metals in the mentioned soil, the limit value of the Cd content (according to the legislation valid in the Slovak Republic, Act number 220/2004) was exceeded 2.34 times and represented the value of 1.64 mg/kg. Additionally, the threshold value given by the European Commission (EC) (2006) was exceeded. Other analyzed heavy metals in soil did not exceed the limit values. The presence and toxicity of risk elements in the soil is a major threat, as they can seriously damage human health through the food chain [67].

The nature of soil contamination can be described by factors such as the contamination factor ($C_{f}^{i}$ and degree of contamination (C_deg_) [30]. The average $C_{f}^{i}$ values for heavy metals (Mn, Zn, Cr, Cu, Ni, Cd, Pb and Hg) are in the range from 0.16 (Hg) to 16.4 (Cd). Based on cf values, we can state that the soil is weakly contaminated by Mn, Cr, Ni, Pb, and Hg ($C_{f}^{i}$ < 1), moderately contaminated by Zn and Cu (1 < $C_{f}^{i}$ < 3) and very highly contaminated by Cd ($C_{f}^{i}$ > 6). The degree of contamination (C_deg_) is another parameter that indicates the degree of soil contamination by all risk elements. The degree of contamination for our land is 21.31. The monitored soil can be characterized as the soil with the highest degree of contamination (C_deg_ ≥ 20) [57].

We also determined the potential ecological risk factor ($E_{r}^{i}$) for each monitored heavy metal. This parameter is related not only to the concentration of the given risk element in the soil but also to the toxicity of each element. The values determined on the basis of the total content of risk element are given in Table 3. All monitored heavy metals except Cd had this parameter less than 40 ($E_{r}^{i}$ < 40). For Cd, this parameter was 492. Based on these values, we can characterize the soil as highly contaminated with Cd and weakly contaminated with other heavy metals (Mn, Zn, Cr, Cu, Ni, Pb and Hg) [57,58].

Based on the index of geoaccumulation (I_geo_), soils are classified into seven classes [59,68]. The average values of the I_geo_ of the monitored soil were in the range of −3.23 to 3.45. The soil sample had an I_geo_ value < 0 (background values for, Mn, Zn, Cr, Cu, Ni, Cd, Pb, Hg) and a value to I_geo_ ˃ 3 (moderately contaminated for Cd).

The contamination of soil with heavy metals is assessed in terms of the risks arising from possible penetration into plants and the whole food chain. Bioavailable forms of heavy metals in the soil are very important for the assessment and prognosis of crop contamination. The high availability of heavy metals threatens the environment more than their total soil content [69]. Heavy metals can migrate into soil ecosystems and subsequently seriously damage human and animal health. The mobility and bioavailability of heavy metals depend on the chemical, physical, biological and microbiological properties of the soil. Factors that affect the bioavailability of metals include pH, and organic matter content [70,71]. It also depends on the mechanisms of absorption, the oxidation state of the metals present, soil cation exchange capacity, nature, the presence of other nutrients in the soil, soil salinity, but also the climate, which can significantly affect the availability of metals in the soil [68,72,73]. The phytotoxicity of heavy metals is manifested mainly on acidic soils. Plants that are short, less than a meter in length and have a large surface leaf, are more sensitive to the absorption of heavy metals [74]. The content of bioavailable forms of heavy metals in the soil from the crop growing locality Imeľ is presented in the Table 4.

Of the monitored bioavailable forms of heavy metals specified in 1 M NH_4_NO_3_, the critical value according to the legislation valid in the Slovak Republic (Act number 220/2004) was exceeded for Cd and Pb. We can state that high concentrations of bioavailable forms of Cd and Pb are risky. In the case of Cd, the critical value was exceeded 1.2 times, and in the case of Pb, this value was exceeded 2.6 times.

The contents of Zn, Cu and Ni in their bioavailable forms were lower than the critical values set for these elements. Food crops as well as peppers can absorb individual elements from contaminated soil, wastewater used for irrigation as well as from sediments on various parts of vegetables from a polluted environment, which can lead to food contamination and consequently significant risks to human health [75].

For this reason, it is very necessary to carry out constant monitoring of heavy metals in edible parts of plants. Due to the frequent consumption of vegetables, it is very necessary to ensure that the level of these contaminants meets the agreed requirements. The levels of heavy metals (Mn, Zn, Cr, Cu, Ni, Cd, Pb and Hg) in the chili pepper samples are shown in Table 5 and Table 6.

### 3.2. Concentration of Heavy Metals in Plant Samples

Of the essential heavy metals required by the human body in certain low concentrations, we monitored Mn, Zn and Cu in selected varieties of chili peppers. The determined values of Mn content ranged from 1.26 to 2.73 mg/kg FW and the values of Zn content were determined from 1.12 to 2.73 mg/kg FW. The Food Codex of the SR as well as Commission Regulation 1881/2006 (EC) do not specify a limit value for Mn. The limit value for the Zn content in none of the analyzed pepper varieties was exceeded. Assuming that a 70 kg adult consumes 1 g of chili pepper with the highest Zn content (0.42 mg/kg) per day, the daily intake of Zn from chili peppers is 0.00273 mg, which is below the PTMI (21–70 mg/70 kg).

Some of heavy metals such as Cu, Co, Fe, Ni, Mn, Mo, Cr, Se, Mg and Zn have functional roles which are essential for various diverse physiological and biochemical activities in the body and may result in deficiency diseases or syndromes if there are inadequate amounts in the body but in large doses they may cause acute or chronic toxicities [76]. 

Mn, Zn and Cu play an important role in enzymatic reactions as well as in cell growth, regulating tissue development, and playing a role in the body’s immunity. Zn is the only metal that occurs in all classes of enzymes [77]. Cu is the third most common trace element in the body and is an important nutrient in food [78]. Mn is an essential element for humans, but high levels of Mn have been found to cross the placenta during pregnancy, which can affect fetal development [79]. The Cr content in the monitored pepper varieties was recorded in the range from 0.11 to 0.23 mg/kg FW. In the case of this monitored metal from eight varieties of the monitored pepper in three varieties (Sigaretta di Bergamo, Candlelight, Bhut Jolokia White) the limit value (0.2 mg/kg) was exceeded, which is stated in Decree no. 2/1994 Coll. Decree of the Ministry of Health of the Slovak Republic, and in one variety (Scotch Bonet Yellow) the value of Cr was set at the level of the limit value. According to other authors, Cr content in *Capsicum* varieties ranged from 0.03 to 1 mg/kg. Due to industrial processes in the environment, large amounts of Cr compounds can be present, which can have adverse biological and ecological effects. Higher Cr content in plants degrades pigment, reduces carotene and chlorophyll content, changes the membrane structure in plants and induces oxidative stress [80,81]. Cr is presented in the environment mainly in two valence states (VI^+^, III^+^). Hexavalent Cr is more dangerous than trivalent Cr, as it can have toxic effects on humans, animals and plants. Intoxication by this metal can may occur by the consummation of contaminated drinking water, as well as the inhalation of Cr compounds. Genotoxic effects of Cr lead to DNA damage, oxidative stress, and other damage. [82,83]. The determined values of Cu content ranged from 0.70 to 1.75 mg/kg FW and the values of Ni content were determined from 0.07 to 0.23 mg/kg FW. We can state that neither in the case of Cu nor in the case of Ni were the limit values exceeded in the monitored pepper varieties. Assuming that a 70 kg adult consumes 1 g of chili pepper with the highest Cu content (1.75 mg/kg) per day, the daily intake of Cu from chili peppers is 0.00175 mg, which is below the PTMI (35 mg/70 kg). Other authors reported higher content of Cu and Ni content in *Capsicum* varieties (8.8–101 mg/kg DM 1.04–3.47 mg/kg DM, respectively) [43]. In general, Cu has low mobility in plants compared to other elements. Toxicity and excess of dietary Cu is also affected by the chemical form as well as by the interaction with other minerals ingested in the diet. The toxicity of Ni has become a subject of interest due to its increased industrial use. Ni is a micronutrient for some higher plants, its low concentration improves plant growth, but at high concentrations it is toxic to plants [84].

Cultivated crops are the main source of Cd and Pb to the human body. Cd is one of the metals that can significantly degrade the quantity and quality of crops grown, and often exceeds limit values in crops, as it is a significant anthropogenic pollutant [85]. The values of Cd content determined in the monitored varieties of *Capsicum* ranged from 0.010 to 0.082 mg/kg FW. We can state that in the three monitored varieties, the limit value was exceeded according to Decree no. 2/1994 Coll. Decree of the Ministry of Health of the Slovak Republic and values according to the Commission Regulation (EC) (1881/2006). The highest exceedance of the limit value was recorded in the variety Bhut Jolokia White (1.64-fold), followed by the variety Sigaretta di Bergamo (1.30-fold) and a 1.1-fold exceedance of the limit value was recorded in the variety Candlelight. Assuming that a 70 kg adult consumes 30 g of chili pepper with highest Cd content (0.08 mg/kg) per month, the monthly intake of Cd from chili peppers is 0.0024 mg, which is below the PTMI (1.75 mg/70 kg). Other authors reported slightly lower values of Cd content in *Capsicum* varieties, in the range from 0.013 to 0.041 mg/kg FW, and from 0.018 to 0.088 mg/kg FW [86,87].

According to the International Agency for Research on Cancer (IARC), Cd is a Group 1 carcinogen for humans and is associated with the incidence of lung cancer [88]. Heavy metals have the ability to replace important elements in human body, e.g., Cd has an atomic structure very similar to that of Zn, and fits almost perfectly into the Zn binding sites of important enzymes such as RNA transferase, carboxypeptidase and alcohol dehydrogenase [89]. Cd is a very toxic element, and the contamination of the food chain with soil Cd poses a threat to human health. Cd in plants inhibits root growth and seed germination. Many studies have shown that the genotoxicity of Cd is directly related to its effect on DNA structure and function [90].

When evaluating the Pb content in the monitored pepper varieties, we recorded the values in the interval from ND to 0.42 mg/kg FW and we can state that in five varieties the limit value was exceeded (0.10 mg/kg), which is stated in the Commission Regulation (EC) (1881/2006) and in one variety the value of Pb content was at the level of the limit value. The highest value of Pb content was recorded in the variety Bhut Jolokia White, where this elevation was 4.2 times. Assuming that a 70 kg adult consumes 30 g of chili pepper with the highest Pb content (0.42 mg/kg) per month, the monthly intake of Pb from chili peppers is 0.0126 mg, which is below the PTMI (1.75 mg/70 kg). Compared to our results, other authors reported lower values of Pb content (0.007–0.023 mg/kg) in their monitored chili pepper varieties [54]. The content of Hg in neither variety (*CapsicumC*. *annuum* and *CapsicumC*. *chinense*) exceeded the established limit value. Assuming that a 70 kg adult consumes 7 g of chili pepper with the highest Hg content (0.00045 mg/kg) per week, the weekly intake of Hg from chili peppers is 0.00000315 mg, which is below the PTWI (0.28 mg/70 kg). The bioaccumulation factor (BAF) in pepper fruits showed low values (Table 5), and do not reflect the intensity of soil contamination by the determined heavy metals and indicate that the studied species of the genus Capsicum did not accumulate determined heavy metals. The processes of absorption, accumulation and transfer of heavy metals into plants are controlled by the bioavailability of metals in soils [91]. The accumulation of heavy metals in plants can lead to potential toxic effects. When plants die, the tissues break down, the heavy metals absorbed into the plants are redistributed and the soil can be enriched with these metals. Soil to plant transfer is one of the main mechanisms of human exposure to metals through food chain [92].

Therefore, it is very important to link soil properties with the phytotoxicity of monitored heavy metals in soil–crop systems, as this is one of the key steps in assessing and improving the risk of heavy metals in soil [93]. In addition to soil, the plant phytotoxicity is also affected by the plant itself, the species, the varieties, the state of development, the metabolic requirements of the plants as well as the possible abiotic stresses that may occur [94].

### 3.3. Statistical Analysis

#### 3.3.1. Analysis of Variance

**Figure 2:**
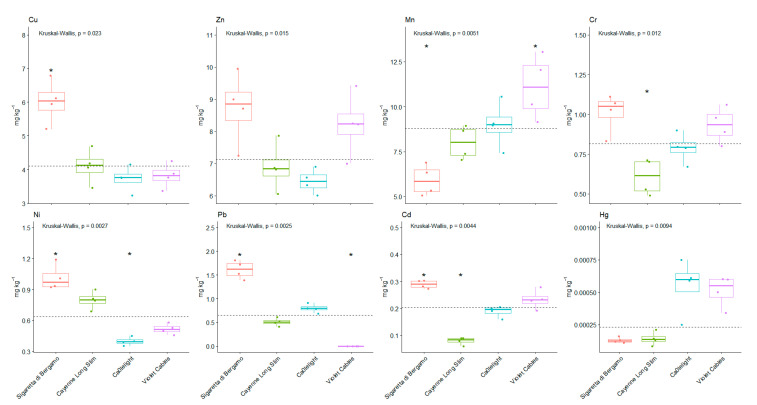
Differences in content of heavy metals in monitored varieties of C. *annuum*. (*) indicates a significantly higher or lower values than the median.

As shown in Figure 2, statistically significant differences were found for the Cd, Ni, and Pb content in the studied *Capsicum annum* cultivars. For Mn content, statistically significant differences were found between SDB and Cayenne, SDB and Candlelight, SDB and Violet, and between Violet and Cayenne and Violet and Candlelight. For Zn content, significant differences were found between SDB and Cayenne, SDB and Candlelight, between Violet and Cayenne and Violet and Candlelight. For Cr content, significant differences were found between SDB and Cayenne, SDB and Candlelight, between Violet and Cayenne, Violet and Candlelight, and between Cayenne and Candlelight. For Cu content, significant differences were found between SDB and Cayenne, Candlelight and Violet Cables. For HG content, significant differences were found between SDB and Candlelight and Violet, and between Cayenne and Candlelight and Violet Cables.

**Figure 3:**
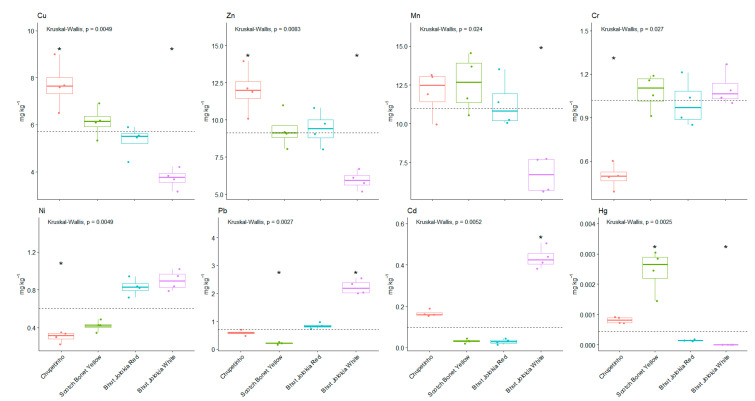
Differences in content of heavy metals in monitored varieties of C. *chinense*. (*) indicates a significantly higher or lower values than the median.

As shown in Figure 3, statistically significant differences were found for the Cu, Pb, and Hg content in the studied *Capsicum chinense* cultivars. For Mn content, significant differences were found between Bhut Jolokia white and other cultivars. For Zn content, significant differences were found between Chupetinho and Scotch Bonet Yellow, BJR and BJW, between BJW and BJR, CH, SBY. For Cr content, significant differences were found between Chupetinho and other cultivars. For Ni content, significant differences were found between Chupetinho and other cultivars, and between Scotch Bonet Yellow and other cultivars. For Cd content, significant differences were found between Chupetinho and other cultivars, and between BJW and other cultivars.

#### 3.3.2. The Relationships between the Tested Parameters

**Figure 4:**
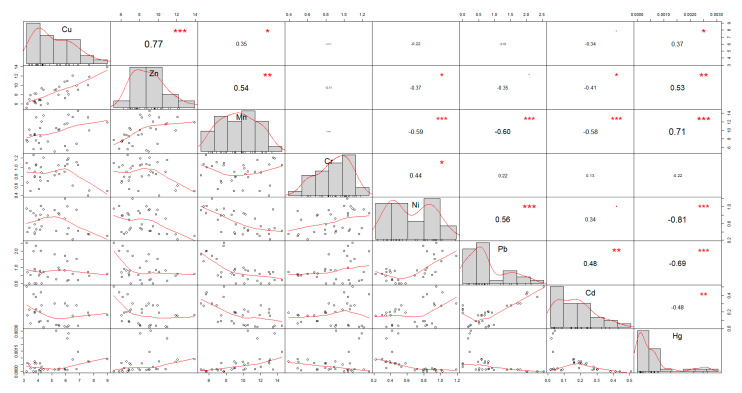
The relationships between the tested parameters. (*)—weak correlation, (**)—moderate correlation, (***)—strong correlation.

In order to examine the mutual relations among the analyzed parameters (total contents of heavy metals), the linear regressions were obtained. The results are shown in Figure 4.

The statistical evaluation of the results confirmed a strong correlation between Cu and Zn content (r = 0.77), Ni and Pb content (r = 0.56), and Mn and Hg content (r = 0.71), and a strong negative correlation between Mn and Ni content (r = −0.59), Mn and Pb content (r = −0.60), Mn and Cd content (r = −0.58), Ni and Hg content (r = −0.81), and Pb and Hg content (−0.69).

#### 3.3.3. Principal Component Analysis

**Figure 5:**
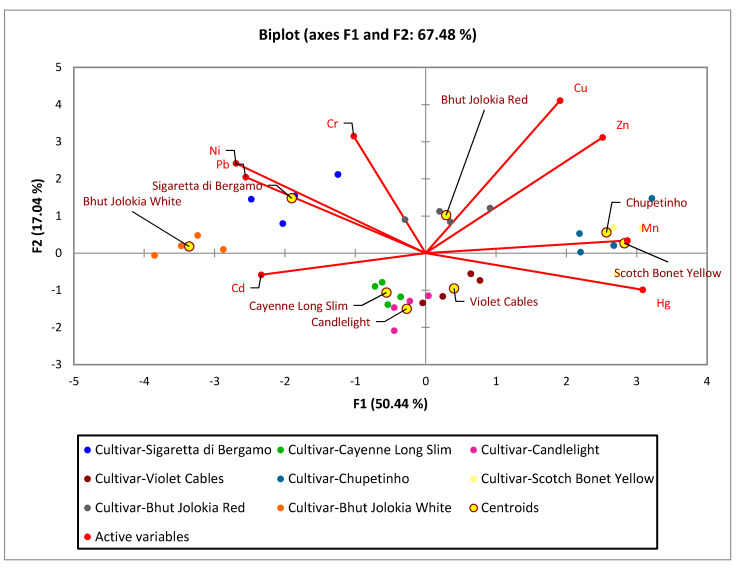
Plot of the first two PC loadings.

To find hidden patterns between the tested parameters, the Spearman PCA was performed. The results of the Bartlett’s test of sphericity indicate that the data were likely factorable (Chi-square (observed = 156.039), Chi-square (critical = 49.801), *p* ≤ 0.0001). The principal component analysis revealed that 67.48% of the total variability embodied in eight parameters could be effectively condensed into and explained by the first two principal components, with eigenvalues of 4.04 and 1.36, respectively. The remaining principal components had eigen values below 1; therefore, they were not suitable for PCA analysis. The F1, accounting for 50.44% of inertia, was represented by Hg, Pb, Ni, Mn and Zn contents. F2 explained 17.04% of the inertia represented mostly by Cu content. The observations are represented by their projections, and the variables are represented by their correlations in Figure 4. As can be seen in Figure 5, cultivars Chupetinho and Scotch Bonet Yellow are clearly characterized by Mn and Hg contents, while Sigaretta di Bergamo is characterized by Ni and Pb contents. The results of the PCA analysis also show that the Cayenne Long Slim, Candlelight and Violet Cables cultivars are similar. The cultivar Bhut Jolokia White is characterized by a high Cd content.

## 4. Conclusions

In southern Slovakia, the interest in growing chili peppers has been increasing recently, due to their benefits. Monitoring soil hygiene is very important, as there is a transport, transformation, or accumulation of potentially hazardous substances in the soil, which can then pass into the cultivated above-ground matter and thus seriously endanger the health of the consumer.

Our achieved results suggest that based on the contamination factor and ecological risk factor, we can characterize the soil as highly contaminated with Cd ($C_{f}^{i}$ = 16.4, = 492) and weakly contaminated with other heavy metals (Mn, Zn, Cr, Cu, Ni, Pb, and Hg). The limit values of bioavailable forms of Cd and Pb in soil, set by Act no. 220/2004 (valid in the Slovak Republic), were exceeded. The low bioaccumulation factor (BAF) in chili pepper fruits did not reflect the intensity of soil contamination by the determined heavy metals and indicate that the studied species of the genus Capsicum do not accumulate monitored heavy metals (Mn, Zn, Cr, Cu, Ni, Cd, Pb, Hg).

We can conclude that the monitored varieties of chili peppers in terms of content and the accumulation of heavy metals, also being based on health-based guidance values (PMTDI, PTMI, PTWI), do not pose a health risk to the consumer. Variety is one of the main factors that can affect the accumulation of heavy metals. The results show that the individual varieties of selected chili peppers did not equally accumulate the monitored heavy metals.

Pb accumulated most in Sigaretta di Bergamo (C. *annuum*) and the least in Scotch Bonnet Yellow (C. *chinense*). Cd accumulated the most in Bhut Jolokia White (C. *chinense*) and the least in Bhut Jolokia Red and Scotch Bonnet Yellow (C. *chinense*). The results of the PCA analysis show that cultivars Chupetinho and Scotch Bonet Yellow are characterized by Mn and Hg contents, while Sigaretta di Bergamo is characterized by Ni and Pb contents. The cultivar Bhut Jolokia White is characterized by a high Cd content. The results obtained can be a challenge for both the agricultural and breeding sector to grow and breed the most suitable varieties in terms of the accumulation of heavy metals and thus prevent their entry into the food chain.

## Figures and Tables

**Table 1:** Validation parameters for determination of the elements.

Element	Wavelength (nm)	LoD (mg/L)	LoQ (mg/L)	Recovery (%)
Mn	279.5	0.0250	0.0300	102.4
Zn	213.9	0.0870	0.1740	106.2
Cu	324.8	0.0876	0.0911	99.6
Cr	359.7	0.0300	0.050	92.2
Ni	232.0	0.3576	0.5646	104.9
Pb	217.0	0.0894	0.1411	93.7
Cd	228.8	0.0621	0.1200	91.6
Hg	253.7	0.02 *	0.04 *	97.1

* LoD limit of detection, LoQ limit of quantification, LoD and LoQ values for Hg expressed as nanograms per sample. Recovery was calculated for quality check of the solutions of known concentrations.

**Table 2:** Agrochemical characteristic of soil of the cultivation area in mg/kg (as a mean of 4 realized analyses, no samples were excluded).

	Ca	K	Mg	P	N_an_	pH/CaCl_2_	Humus (%)	C_OX_ (%)
Min	3879	139.15	305.68	168	43.9	7.47	3.06	1.77
Max	4150	147.12	338.15	180	46.34	7.63	3.17	1.83
Median	3975.5	143.065	316.46	173	45.08	7.55	3.085	1.8
Average	3995	143.1	319.19	174	45.1	7.55	3.1	1.8
STDEV	112.91	3.28	13.95	5.67	1.21	0.07	0.05	0.03

**Table 3:** Total content of heavy metals (Ci—CMn, CZn, CCr, CCu, CNi, CCd, CPb, CHg) in soil in mg/kg (as a mean of 4 realized analyses, no samples were excluded).

	Mn	Zn	Cr	Cu	Ni	Cd	Pb	Hg
Minimum	333.65	77.5	7.52	28.88	12.92	1.59	13.89	0.0125
Maximum	345.23	79.1	7.90	30.78	13.73	1.68	14.7	0.0132
Median	341.76	78.1	7.69	29.98	13.27	1645	14.29	0.0128
Average	340.6	78.2	7.7	29.9	13.3	1.64	14.30	0.013
STDEV	4.95	0.66	0.16	0.79	0.36	0.037	0.42	0.0003
Contamination factor ($C_{f}^{i}$)	0.85	1.10	0.22	1.19	0.67	16.4	0.72	0.16
Potential ecological risk factor ($E_{r}^{i}$)	0.85	1.10	0.44	5.98	3.33	492	3.57	6.41
Geoaccumulation Index (I_geo_)	−0.82	−0.44	−2.77	−0.33	−1.17	3.45	−1.07	−3.23
Limit value		150		60.0	50.0	0.70	70	0.50
Threshold value		100		40.00	30.0	0.50	50	

Limit value—for a soil extract by *aqua regia* according to legislation valid in the Slovak Republic (Act number 220/2004), threshold value—European Commission (2006).

**Table 4:** Content of bioavailable forms of heavy metals (Mn, Zn, Cr, Cu, Ni, Cd, Pb) in soil in mg/kg (as a mean of 4 realized analyses, no samples were excluded).

	Mn	Zn	Cr	Cu	Ni	Cd	Pb
Minimum	0.14	0.18	0.03	0.19	0.14	0.1	0.24
Maximum	0.18	0.21	0.07	0.24	0.18	0.14	0.28
Median	0.16	0.185	0.05	0.225	0.16	0.12	0.26
Average	0.16	0.19	0.05	0.22	0.16	0.12	0.26
STDEV	0.018	0.014	0.018	0.022	0.018	0.018	0.018
Critical value		2.00		1.00	1.50	0.10	0.10

Critical value—for a soil extract by NH_4_NO_3_ according to legislation valid in the Slovak Republic (Act number 220/2004).

**Table 5:** Total content of heavy metals in *Capsicum* samples in mg/kg fresh weight (as a mean of 4 realized analyses, no samples were excluded).

mg/kg FW	Mn	Zn	Cr	Cu	Ni	Cd	Pb	Hg
*C*. *annuum*								
1. Sigaretta di Bergamo	1.32 ± 0.19	1.95 ± 0.25	0.23 ± 0.03	1.35 ± 0.15	0.23 ± 0.03	0.06 ± 0.003	0.36 ± 0.04	0.00003 ± 0.000005
MINIMUM	1.13	1.62	0.19	1.16	0.21	0.061	0.31	0.000025
MAXIMUM	1.54	2.23	0.25	1.52	0.27	0.068	0.41	0.000036
MEDIAN	1.31	1.98	0.24	1.35	0.22	0.065	0.36	0.000028
BAF	0.017	0.112	0.131	0.201	0.076	0.177	0.112	0.01
2. Cayenne Long Slim	1.61 ± 0.19	1.39 ± 0.15	0.12 ± 0.02	0.82 ± 0.10	0.16 ± 0.02	0.02 ± 0.003	0.10 ± 0.02	0.00003 ± 0.00001
MINIMUM	1.42	1.22	0.10	0.70	0.14	0.012	0.08	0.000017
MAXIMUM	1.79	1.58	0.14	0.94	0.18	0.018	0.12	0.000042
MEDIAN	1.61	1.37	0.12	0.83	0.16	0.017	0.10	0.000028
BAF	0.024	0.088	0.079	0.138	0.06	0.049	0.036	0.011
3. Candlelight	2.73 ± 0.34	2.74 ± 0.36	0.11 ± 0.02	1.75 ± 0.23	0.07 ± 0.01	0.04 ± 0.004	0.13 ± 0.02	0.00018 ± 0.00002
MINIMUM	2.27	2.30	0.09	1.48	0.05	0.035	0.11	0.000161
MAXIMUM	2.99	3.18	0.14	2.04	0.08	0.043	0.16	0.000207
MEDIAN	2.84	2.73	0.11	1.74	0.07	0.036	0.13	0.000184
BAF	0.026	0.082	0.103	0.125	0.03	0.116	0.056	0.042
4. Violet Cables	2.24 ± 0.36	1.66 ± 0.20	0.19 ± 0.02	0.77 ± 0.07	0.10 ± 0.01	0.05 ± 0.007	ND	0.0001 ± 0.00003
MINIMUM	1.84	1.41	0.16	0.68	0.09	0.039	ND	0.000069
MAXIMUM	2.63	1.90	0.21	0.86	0.12	0.056	ND	0.000121
MEDIAN	2.24	1.66	0.19	0.77	0.10	0.047	ND	0.000111
BAF	0.033	0.105	0.121	0.128	0.039	0.140	ND	0.039
*C*. *chinense*								
5. Chupetinho	2.34 ± 0.34	1.73 ± 0.23	0.20 ± 0.02	1.14 ± 0.12	0.08 ± 0.01	0.01 ± 0.002	0.04 ± 0.01	0.00045 ± 0.00013
MINIMUM	1.95	1.49	0.17	0.99	0.06	0.004	0.03	0.000269
MAXIMUM	2.70	2.04	0.22	1.28	0.09	0.008	0.05	0.000566
MEDIAN	2.35	1.69	0.20	1.14	0.08	0.006	0.04	0.000491
BAF	0.035	0.160	0.065	0.257	0.023	0.098	0.041	0.061
6. Scotch Bonnet Yellow	2.64 ± 0.38	1.89 ± 0.11	0.23 ± 0.03	1.09 ± 0.11	0.12 ± 0.01	0.06 ± 0.006	0.23 ± 0.03	0.00016 ± 0.00006
MINIMUM	2.18	1.76	0.20	0.95	0.10	0.047	0.20	0.000073
MAXIMUM	3.10	2.03	0.26	1.22	0.13	0.060	0.27	0.000220
MEDIAN	2.64	1.89	0.23	1.10	0.12	0.058	0.23	0.000176
BAF	0.037	0.119	0.140	0.205	0.032	0.018	0.015	0.188
7. Bhut Jolokia Red	2.01 ± 0.28	1.68 ± 0.21	0.18 ± 0.03	0.95 ± 0.12	0.15 ± 0.02	0.01 ± 0.002	0.15 ± 0.02	0.00003 ± 0.000004
MINIMUM	1.79	1.43	0.15	0.78	0.13	0.002	0.13	0.000020
MAXIMUM	2.41	1.93	0.22	1.05	0.17	0.008	0.17	0.000031
MEDIAN	1.93	1.68	0.17	0.98	0.15	0.005	0.15	0.000026
BAF	0.030	0.120	0.130	0.178	0.06	0.018	0.058	0.011
8. Bhut Jolokia White	1.26 ± 0.22	1.12 ± 0.12	0.21 ± 0.02	0.70 ± 0.08	0.17 ± 0.02	0.08 ± 0.010	0.42 ± 0.05	0.00012 ± 0.00002
MINIMUM	1.06	0.98	0.19	0.60	0.15	0.072	0.38	0.000103
MAXIMUM	1.46	1.26	0.24	0.79	0.19	0.095	0.48	0.000141
MEDIAN	1.26	1.12	0.20	0.71	0.17	0.080	0.41	0.000122
BAF	0.020	0.076	0.143	0.125	0.068	0.262	0.086	
Limit		10.0	0.2	10.0	0.50	0.05	0.5	0.02
Maximal level						0.05	0.10	
Tolerable intake		PMTDI 0.3–1		PMTDI 0.5		PTMI 0.025	PTMI 0.025	PTWI 0.004

Limit value according to the Food Codex of the Slovak Republic, maximal level according to Commission Regulation 1881/2006(EC), PMTDI—provisional maximum tolerable daily intake (mg/kg body weight/day) according to WHO, PTMI—provisional tolerable monthly intake (mg/kg body weight/month) according to WHO, PTWI—provisional tolerably weekly intake (mg/kg body weight/week) according to WHO. ND—not detected

**Table 6:** Total content of heavy metals in *Capsicum* samples in mg/kg dry matter (as a mean of 4 realized analyses, no samples were excluded).

mg/kg DM	Mn	Zn	Cr	Cu	Ni	Cd	Pb	Hg
*C*. *annuum*								
1. Sigaretta di Bergamo	5.91 ± 0.85 ^a^	8.72 ± 1.11 ^a^	1.01 ± 0.12 ^a^	6.01 ± 0.65 ^a^	1.01 ± 0.13 ^a^	0.29 ± 0.01 ^a^	1.61 ± 0.19 ^a^	0.00013 ± 0.00002 ^a^
MINIMUM	5.06	7.25	0.83	5.20	0.92	0.27	1.39	0.00011
MAXIMUM	6.88	9.94	1.11	6.78	1.19	0.30	1.81	0.000159
MEDIAN	5.85	8.85	1.05	6.04	0.97	0.29	1.62	0.000124
2. Cayenne Long Slim	8.01 ± 0.93 ^b^	6.90 ± 0.75 ^b^	0.61 ± 0.11 ^b^	4.10 ± 0.50 ^b^	0.80 ± 0.09 ^b^	0.08 ± 0.01 ^b^	0.51 ± 0.08 ^b^	0.00014 ± 0.00005 ^a^
MINIMUM	7.05	6.05	0.49	3.46	0.69	0.06	0.41	0.0001
MAXIMUM	8.93	7.87	0.71	4.69	0.90	0.09	0.61	0.000211
MEDIAN	8.02	6.84	0.62	4.13	0.80	0.09	0.51	0.000137
3. Candlelight	9.00 ± 1.28 ^b^	6.45 ± 0.38 ^b^	0.79 ± 0.09 ^c^	3.73 ± 0.38 ^b^	0.40 ± 0.04 ^c^	0.19 ± 0.02 ^c^	0.80 ± 0.09 ^c^	0.00055 ± 0.00021 ^b^
MINIMUM	7.42	6.01	0.67	3.23	0.35	0.16	0.69	0.00025
MAXIMUM	10.56	6.90	0.90	4.15	0.45	0.21	0.91	0.00075
MEDIAN	9.01	6.45	0.79	3.76	0.40	0.20	0.80	0.0006
4. Violet Cables	11.10 ± 1.77 ^c^	8.23 ± 0.98 ^a^	0.93 ± 0.11 ^a^	3.82 ± 0.37 ^b^	0.52 ± 0.05 ^d^	0.23 ± 0.04 ^d^	ND	0.00051 ± 0.00012 ^b^
MINIMUM	9.15	7.01	0.80	3.37	0.46	0.19	ND	0.00034
MAXIMUM	13.05	9.42	1.06	4.26	0.58	0.28	ND	0.000601
MEDIAN	11.10	8.24	0.94	3.83	0.52	0.23	ND	0.000551
BAF	0.033	0.105	0.121	0.128	0.039	0.140	ND	0.039
*C*. *chinense*								
5. Chupetinho	12.01 ± 1.47 ^a^	12.01 ± 1.58 ^a^	0.50 ± 0.09 ^a^	7.69 ± 1.02 ^a^	0.30 ± 0.06 ^a^	0.17 ± 0.02 ^a^	0.59 ± 0.09 ^a^	0.00081 ± 0.00011 ^a^
MINIMUM	9.96	10.09	0.39	6.49	0.22	0.15	0.48	0.000709
MAXIMUM	13.13	13.95	0.60	8.98	0.35	0.19	0.70	0.00091
MEDIAN	12.47	12.01	0.50	7.64	0.32	0.16	0.59	0.000809
6. Scotch Bonnet Yellow	12.60 ± 1.83 ^a^	9.32 ± 1.22 ^b^	1.08 ± 0.13 ^b^	6.13 ± 0.65 ^b^	0.42 ± 0.06 ^b^	0.03 ± 0.01 ^b^	0.21 ± 0.04 ^b^	0.00245 ± 0.00071 ^b^
MINIMUM	10.53	8.05	0.91	5.31	0.35	0.02	0.16	0.00145
MAXIMUM	14.53	10.99	1.19	6.89	0.49	0.04	0.25	0.003049
MEDIAN	12.67	9.13	1.11	6.15	0.42	0.03	0.21	0.002649
7. Bhut Jolokia Red	11.30 ± 1.58 ^a^	9.41 ± 1.17 ^b^	1.00 ± 0.16 ^b^	5.33 ± 0.65 ^c^	0.83 ± 0.09 ^c^	0.03 ± 0.01 ^b^	0.83 ± 0.11 ^c^	0.00014 ± 0.00002 ^c^
MINIMUM	10.05	8.02	0.85	4.39	0.72	0.01	0.72	0.000114
MAXIMUM	13.50	10.81	1.21	5.90	0.94	0.04	0.98	0.000175
MEDIAN	10.83	9.41	0.97	5.51	0.83	0.03	0.82	0.000145
8. Bhut Jolokia White	6.70 ± 1.17 ^b^	5.95 ± 0.63 ^c^	1.10 ± 0.12 ^b^	3.73 ± 0.43 ^d^	0.90 ± 0.10 ^c^	0.43 ± 0.0529 ^c^	2.23 ± 0.26 ^d^	0.00065 ± 0.00008 ^d^
MINIMUM	5.63	5.19	1.00	3.17	0.79	0.382	2.00	0.00055
MAXIMUM	7.74	6.70	1.27	4.21	1.02	0.506	2.54	0.00075
MEDIAN	6.72	5.95	1.07	3.77	0.90	0.4255	2.19	0.000651

Different lowercase letters indicate a significant difference between the treatments. ND—not detected.
